# GP73: the key to unlocking immunotherapies efficacy in solid tumors?

**DOI:** 10.1136/jitc-2025-011989

**Published:** 2025-05-13

**Authors:** Rebecca J Bayliss, Alan L Parker

**Affiliations:** 1 Division of Cancer and Genetics, Cardiff University, Cardiff, UK

**Keywords:** T cell, Tumor microenvironment - TME, Immunotherapy, Biomarker

## Abstract

Resident Golgi protein 73 (GP73) is expressed in many healthy tissues, however overexpression is associated with both viral infections and cancer. As an oncoprotein, GP73 drives tumor progression and plays a fundamental role in immune regulation. A recent publication illustrates a role for GP73 in T-cell antitumor immunity employing GP73 genetically depleted T-cell mouse models. GP73-deficient T-cells were found to detrimentally affect CD8+T cell cytotoxicity and glycolysis primarily due to its interaction with Hypoxia-inducible factor 1α and mTOR levels in hypoxic cells, suggesting a key role for GP73 in T-cell cytotoxicity within the hypoxic tumor microenvironment. This finding opens the door to the potential development of GP73 targeting through ectopic expression of GP73 which was found to restore glycolysis and therefore T-cell cytotoxicity resulting in tumor regression. In addition, GP73 was found to be a potential biomarker to inform clinical treatment of patients undergoing immunotherapy. Could GP73 be the key to establishing a therapeutic strategy for generating improved patient responses to immunotherapy?

 Human Golgi protein 73 (GP73, GOLM1, GOLPH2) is a 73 KDa type II integral transmembrane protein localized to the cis and medial Golgi cisternae. GP73 is well established in cancer and antitumor immunity. Here we highlight a recent publication^1^ exploring the role of GP73 in T-cell cytotoxicity within the hypoxic tumor microenvironment (TME) with conceivable translational potential.

## GP73 in cancer

GP73 is endogenously expressed in a broad range of human epithelial tissues, however overexpression is a key marker of both viral and non-viral liver disease, including hepatocellular carcinoma (HCC), with the cleaved soluble form of GP73 often associated with liver and prostate cancer found in patient sera.^2^

Oncoprotein GP73 is associated with various malignancies including HCC, prostate cancer and glioblastoma with increased expression correlating with enhanced metastatic burden and decreased patient survival. Through multiple interacting partners GP73 is able to enhance the secretion of key cancer-promoting signal proteins including; alpha fetoprotein, immune checkpoint protein B7-H3, and vascular endothelial growth factor (VEGF), leading to increased proliferation, metastasis, angiogenesis, oxidative stress and drug resistance of cancer cells.^3 4^ Furthermore, overexpression of GP73 inhibits DNA binding of p53,^5^ increases cell surface recycling of epidermal growth factor receptor (EGFR) and Receptor tyrosine kinases, affecting the expression of cancer-related proteins downstream such as E-cadherin and metalloproteinases.^6^

## GP73 in antitumor immunity

Due to GP73’s role in protein secretion it is not surprizing it plays a key role in cytokine and chemokine homeostasis. Cytokines and chemokines mediate signaling between cells to tightly regulate the immune system. Cellular proteins including cytokines are trafficked through the Golgi and therefore aberrant cytokine production has been associated with GP73 levels. Increases in GP73 have been linked to a reduction in type 1 interferon (IFN) and other inflammatory cytokines, whereas soluble GP73 inhibits IL-12 production in monocyte-derived dendritic cells suppressing the antitumor response, whereas overexpression promotes chemokine ligand-2 transcription contributing to tumor metastasis and progression through recruitment of myeloid-derived suppressor cells.^4^ Importantly, GP73 is a key driver of hypoxia influencing the TME and tumor immunity by regulating programmed death-ligand 1 (PD-L1) expression through the EGFR/STAT3 signaling pathway in HCC^7^ and endoplasmic reticulum stress in tumors leading to an immunosuppressive microenvironment promoting PD-L1 stabilization, transporting PD-L1 to tumor-associated macrophages (TAMS) and leading to CD+8 T-cell suppression.^8^

Despite the well-established role of GP73 in immune regulation, to date, little has been reported on the role of GP73 in T-cells within the TME. Liu *et al* remedy this by undertaking a novel approach investigating the role of GP73 in T-cell mediated antitumor immunity using genetically depleted T-cells in three mouse models: a highly immunogenic orthotopic MC38 colon tumor cell mouse model, a moderately immunogenic B16-F10 melanoma model and a poor immunogenic LLC model. The study revealed that MC38 tumors grew more rapidly in the presence of GP73-depleted T-cells, whereas B16-F10 saw a moderate increase in growth, with cells more readily metastasized in GP73-depleted T-cells. The authors demonstrate that GP73-depleted macrophages did not influence tumor growth, indicative of a GP73-dependent role in T-cell tumor control. RNA sequencing analysis of the MC38 tumors revealed a role for both hypoxia and epithelial–mesenchymal transition signaling pathways implicating a TME more conductive to tumor progression in the absence of GP73. Interestingly, GP73-deficient T cells did not impact the overall proportion of CD8+T cells or CD4+T cells present in the tumors, though GP73-depleted CD8+T cells population expressed lower levels of cytotoxic cytokines Granzyme B and IFN-γ, which unexpectedly appeared independent of immune checkpoints such as programmed cell death protein-1 (PD-1). Single cell sequence analysis of distinct GP73-deficient CD8+T cell subsets showed a notable decrease in Hypoxia-inducible factor 1α (HIF-1α) and a downregulation of cytotoxic genes, alongside changes in genes associated with hypoxia and glycolysis. As HIF-1α plays a key role in glycolysis they hypothesize that a reduction in glycolysis in the CD8+T cells population is due to a loss of HIF-1α which in turn impacts CD8+T cell cytotoxicity.^1^

## The hypoxic TME and HIF-1α

Hypoxia is associated with resistance to radiation and chemotherapy and associated with poor outcomes regardless of treatment. Hypoxia induces expression of a key transcription factor, HIF-1α, expression of which is linked to poor prognosis and resistance to therapy in multiple tumor types. In the absence of oxygen, HIF-1α binds hypoxia response elements activating expression of multiple hypoxia-response genes including the proangiogenic growth factor, VEGF. In the presence of oxygen, HIF-1α is bound to tumor suppressor Von-Hippel-Lindau proteins, leading to ubiquitination and targeting to proteasomal degradation. HIF-1α expression activates genes that reduce oxygen supply and consumption, facilitating metabolic changes from oxidative phosphorylation to anaerobic glycolysis.^9^

Several subtypes of T-cells are located in the TME, supplying energy using different metabolic mechanisms. Cancer requires high levels of glucose, resulting in inactivation of immune cells, especially T-effector cells. Therefore HIF-1α activation or inactivation is responsible for the amount and type of energy source available and oxygen levels that determine the fate of T-cells in the TME. Extensive cell proliferation of the tumor increases glucose consumption and reduces metabolites. As glucose and oxygen decrease there is an increase in lactate and in turn increase in the acidity of the TME affecting cytokine production, cell proliferation and immunosuppression, reducing effective CD8+T cells in TME, increasing PD-1 levels and metabolism of fatty acids.^10^

Considering this, it seems plausible that GP73 and HIF-1α adopt a synergistic relationship in T-cells, previous literature demonstrates GP73 stabilizes HIF-1α, promoting VEGF transcription and promoting angiogenesis in the TME. Both proteins are involved in oxidative stress and glycolysis of cells, regulating a hypoxic cancer environment and reducing cytotoxicity of CD8+T cells, however very little is known about that interaction within tumor-infiltrating lymphocytes.

Liu *et al* confirm that GP73 and HIF-1α were continuously upregulated in T-cells in a hypoxic environment, conversely neither HIF-1α nor its upstream regulator, mTOR, an essential component in the hypoxia signaling pathway increased in GP73-depleted T-cell populations. Activity of both HIF-1α and mTOR in T-cells was restored through exogenous overexpression of HIF-1α and pharmacological activation of mTOR. Authors therefore investigated if GP73 directly regulates HIF-1α expression by comparing normoxic and hypoxic conditions. In hypoxic conditions GP73-deficient T-cells saw a reduction in expression of HIF-1α and its downstream gene, VEGF-A, in addition to a reduction in glycolytic genes (LDHA and PDK1). Seahorse analysis concluded that GP73 is required for glycolysis of CD8+T cells to enable cytotoxic function under hypoxic conditions. This was evidenced by reduced cytokine production in hypoxic GP73-deficient T-cells. To explore the potential of GP73 as a therapeutic target, authors explored whether ectopic expression of GP73 in GP73-deficient T-cells could restore T-cell glycolysis. Using a lentiviral system to overexpress GP73 in T-cells within their mouse models they observed restoration of HIF-1α, VEGF-A, LDHA and PDK1 levels under hypoxic conditions. Additionally, they observed that glucose uptake of GP73-deficient cells was improved under ectopic induction of GP73 and importantly led to significantly delayed tumor growth. This led to the hypothesis that enhancing GP73 expression in T-cells using an ectopic approach could be beneficial to enhancing antitumor immunity and that the clinical efficacy of immunotherapies may be related to GP73 levels in T-cells.

Finally, the authors sought to demonstrate a correlation between GP73 expression in T-cells from clinical samples and their response to PD-1 immunotherapy. In patients with lung cancer with a partial response to anti-PD-1 the authors observed higher GP73 T-cell levels associated with better anti-PD-1 efficacy indicating potential to use GP73 expression in T-cells as a biomarker to assess patient response to immunotherapy. Therefore targeting GP73 via CAR-T-cell therapy may provide a strategy to enhance the effectiveness of immunotherapies.^1^

## Future directions

The use of immune checkpoint inhibitors to unleash the body’s immune system to attack cancerous tissue has revolutionized anticancer therapy in recent years. Despite efficacy in some tumor types, patient responses in many solid tumor types remain poor. The success of immunotherapy broadly relies on the availability of primed T-cells capable of cytotoxic responses once the checkpoint blockade has been lifted and therefore an immunologically “cold” TME can dampen responses in patients with progressive disease. An aggressive hypoxic environment can further contribute to limited efficacy. Therefore, research into the T-cells present within the TME under hypoxic and immunosuppressive conditions is essential to address such limitations.

The findings of Liu *et al* present a potential solution. The authors propose the development of an adoptive therapy targeting GP73 to encourage CD8+ T cell cytotoxicity to enhance the efficacy of immunotherapy. These findings are certainly appealing and could have the potential to increase the efficacy of immunotherapies to a broader range of tumors. However, from a safety perspective, further research is required in human ex vivo models to answer important outstanding questions. Would this approach be beneficial in a poorly immunogenic tumor type such as pancreatic cancer? Could overexpression of GP73 lead to T-cell exhaustion over prolonged periods exacerbating the already immunosuppressed TME? Was increased anti-PD-1 efficacy due to GP73 presence enhancing PD-1 expression as seen in TAMs, thus making anti-PD-1 better able to bind its target? Will overexpressing GP73 increase PD-1/PD-L1 expression in humans? And how will increased expression in T-cells influence GP73 role in tumors? Nonetheless, these findings give hope of developing more efficacious, longer-lasting immunotherapies with the potential to overcome the hostile TME.

## Supplementary material

10.1136/jitc-2025-011989 online supplemental file 1
