# Supplementary material for: GP73: the key to unlocking immunotherapies efficacy in solid tumors?
Source: J Immunother Cancer. 2025 May 13;13(5):e011989. doi: 10.1136/jitc-2025-011989 (PMC12083270; doi:10.1136/jitc-2025-011989)
Supplement: online supplemental file 1 [file jitc-13-5-s001.pdf]

Graphical Abstract

GP73 reinforces cytotoxic T-cell function by regulating HIF-1α and increasing antitumor efficacy.

Liu J, Feng C, Zhao R, Song H, Huang L, Jiang N, Yang X, Liu L, Duan C, Wan L, Gao Q, Sun L, Hou X, Liu M, Zhang Y, Zhang X, Zhang D, Wang Y, Li Y, Ma X, Zhong H, Min M, Wei C, Cao Y, Yang X

- GP73 depleted T-cells (GP73-ve T-cells, pink) display reduced expression of HIF-1α and mTOR resulting in loss of glycolytic function and reduced cytotoxicity of CD8+ T-cells and therefore enhanced tumor growth.

- Introduction of endogenous GP73 (GP73+ve T-cell, green) restores HIF-1α and mTOR expression increasing glycolysis and cytotoxic cytokine production in CD8+ T-cells leading to tumor regression.

- Enhanced cytotoxic function of T-cells within the hypoxic TME has potential as a novel strategy to enhance effectiveness of immunotherapies in solid tumors.

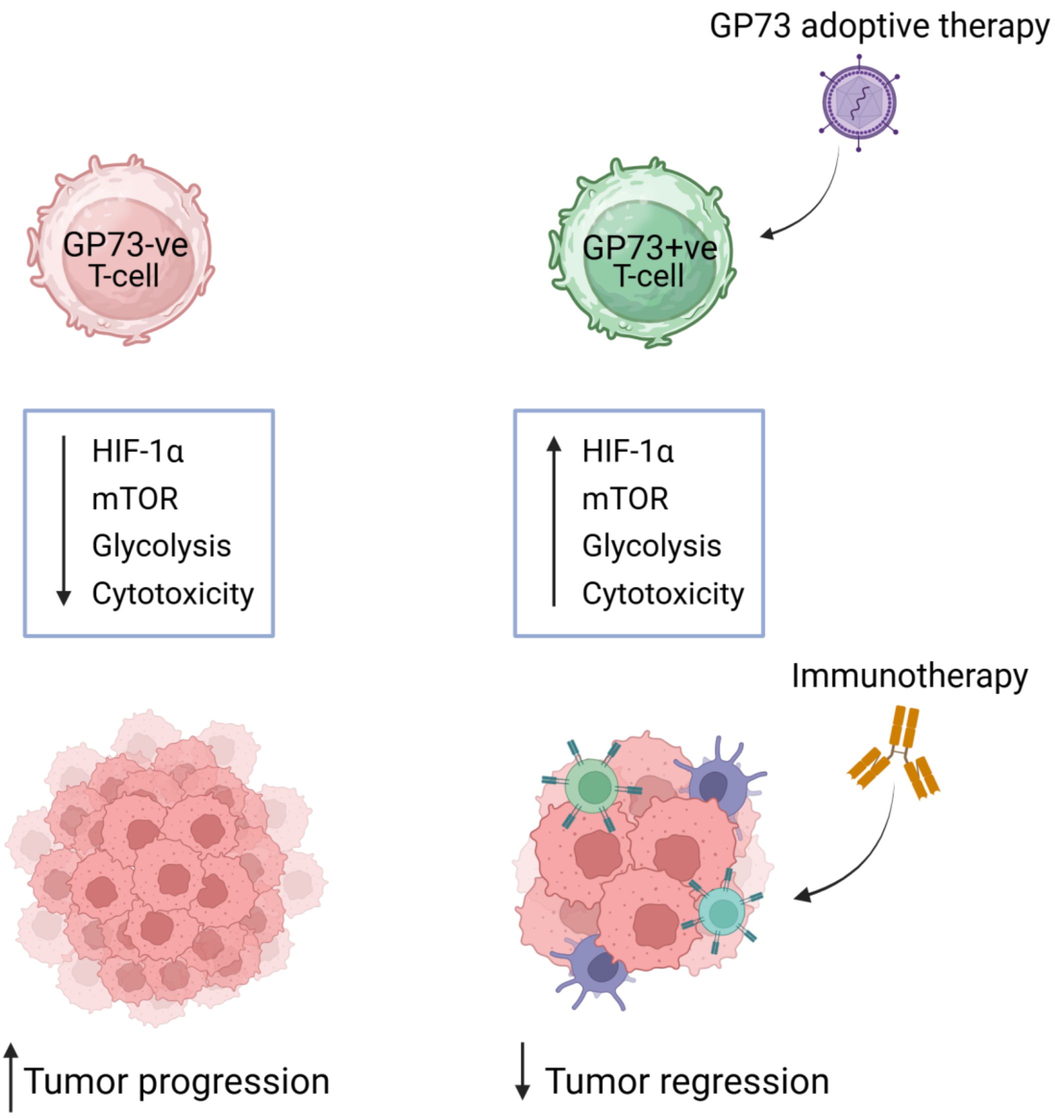

Created in BioRender. Parker, A. (2025)
https://BioRender.com/m5cg9c6
